# *Sarocladium* and *Lecanicillium* Associated with Maize Seeds and Their Potential to Form Selected Secondary Metabolites

**DOI:** 10.3390/biom11010098

**Published:** 2021-01-13

**Authors:** Lidia Błaszczyk, Agnieszka Waśkiewicz, Karolina Gromadzka, Katarzyna Mikołajczak, Jerzy Chełkowski

**Affiliations:** 1 Institute of Plant Genetics, Polish Academy of Sciences, Strzeszyńska 34, 60-479 Poznań, Poland; kmiko@igr.poznan.pl (K.M.); jche@igr.poznan.pl (J.C.); 2 Department of Chemistry, Poznan University of Life Sciences, Wojska Polskiego 75, 60-625 Poznań, Poland; agnieszka.waskiewicz@up.poznan.pl (A.W.); karolina.gromadzka@up.poznan.pl (K.G.)

**Keywords:** maize seed-associated fungi, *Sarocladium zeae*, *Sarocladium strictum*, *Lecanicillium lecanii*, mycotoxins, bioactive compounds

## Abstract

The occurrence and diversity of *Lecanicillium* and *Sarocladium* in maize seeds and their role in this cereal are poorly understood. Therefore, the present study aimed to investigate *Sarocladium* and *Lecanicillium* communities found in endosphere of maize seeds collected from fields in Poland and their potential to form selected bioactive substances. The sequencing of the internally transcribed spacer regions 1 (ITS 1) and 2 (ITS2) and the large-subunit (LSU, 28S) of the rRNA gene cluster resulted in the identification of 17 *Sarocladium zeae* strains, three *Sarocladium strictum* and five *Lecanicillium lecanii* isolates. The assay on solid substrate showed that *S. zeae* and *S. strictum* can synthesize bassianolide, vertilecanin A, vertilecanin A methyl ester, 2-decenedioic acid and 10-hydroxy-8-decenoic acid. This is also the first study revealing the ability of these two species to produce beauvericin and enniatin B1, respectively. Moreover, for the first time in the present investigation, pyrrocidine A and/or B have been annotated as metabolites of *S. strictum* and *L. lecanii*. The production of toxic, insecticidal and antibacterial compounds in cultures of *S. strictum, S. zeae* and *L. lecanii* suggests the requirement to revise the approach to study the biological role of fungi inhabiting maize seeds.

## 1. Introduction

The seeds of many agronomically important crops such as maize (*Zea maize* L.) are frequently colonized by fungal communities, both externally and internally. Recent studies on the composition and variation in the seed-associated fungal mycobiomes have shown *Aspergillus*, *Alternaria*, *Cladosporium*, *Curvularia*, *Fusarium*, *Mucor*, *Penicillium*, *Sarocladium*, and *Trichoderma* to be the most prevalent fungal taxa in Ascomycota [1,2,3]. In Basidiomycota, *Wallemia* was the main genus observed to be associated with crop seeds [3]. *Aspergillus*, *Fusarium*, *Wallemia*, *Sarocladium*, and *Penicillium* were also reported as the predominant genera occurring in various maize kernel storage conditions [3]. At the species level, *Cladosporium sphaerospermum*, *Penicillium aurantiogriseum*, and *Trichoderma gamsii* were exclusively isolated from the internal tissue of maize seeds [2]. *Aspergillus flavus*, *Aspergillus penicillioides*, *Aspergillus niger*, *Cladosporium cladosporioides*, *Fusarium andiyazi, Fusarium graminearum, Fusarium incarnatum-equiseti, Fusarium nygamai, Trichoderma longibrachiatum*, *Trichoderma harzianum, Penicillium digitatum, Mucor fragilis*, and *Wallemia sebi* were isolated only from the surface of seeds [1,2,3]. However, *Alternaria alternata*, *Fusarium verticillioides*, *Fusarium proliferatum*, *Penicillium oxalicum*, *Penicillium polonicum*, and *Sarocladium zeae* were isolated from both the external surface and internal tissues [1,2,3]. The occurrence of *F. verticillioides* and *S. zeae* in maize seeds has also been reported in our previous study [4]. Moreover, our study documented the presence of *Fusarium subglutinans*, *Trichoderma atroviride*, and *Lecanicillium lecanii* [4]. These species were identified on average from 1 to 35% in maize ear samples with *Fusarium* maize ear rot (MER) symptoms.

Most of the maize seed-associated fungi are classified as commensals with yet unknown functions in plants or as pathogens that include toxigenic *Fusarium* and *Aspergillus* species [1,2,3]. The less common ones are those shown to have beneficial effects on plants, such as *Trichoderma*, which is a well-known important Microbial Biological Control Agent (MBCA) that can protect maize plants and enhance their vegetative growth [5]. Several studies have reported a seed-borne protective endophyte of maize, namely *Sarocladium zeae,* as *Acremonium zeae* [6,7,8], which produces dihydroresorcylide and pyrrocidine metabolites that are antagonistic to *A. flavus* and *F. verticillioides* [6,7] as well as hydrolytic enzymes such as amylases, proteases, cellulases, and lipases [1]. However, knowledge about the role of endophytic *Sarocladium* species, including *S. zeae* or *Sarocladium strictum*, in maize is still scarce. There is accumulating evidence that entomopathogenic, nematophagous *Lecanicillium* spp. may demonstrate activity against fungal plant pathogens [9,10]. However, to the best of our knowledge, no studies have investigated how the seed-associated *Lecanicillium* species interact with maize plants. The occurrence and diversity of both *Lecanicillium* and *Sarocladium* in maize seeds and their role in maize plants are still poorly understood. In addition, the potential of these maize seed-borne fungi to produce toxic and/or insecticidal and antibacterial compounds has not yet been demonstrated. Therefore, to gain insights into their diversity, including their ability to produce secondary metabolites, the present study aimed to investigate *Sarocladium* and *Lecanicillium* communities found in maize ears collected from fields before harvest in Poland and their potential to form selected bioactive substances. This could open the way to understanding the functions of endogenous *Sarocladium* and *Lecanicillium* species and indicates a strategy for selecting potential candidates for the biological protection of maize crops.

## 2. Materials and Methods

### 2.1. Fungal Collection

The 17 *S. zeae* strains, three *S. strictum* strains, and five *L. lecanii* strains investigated in the present study are listed in Table 1. Seven *S. zeae* strains, one *S. strictum* strain and five *L. lecanii* strains sourced from maize ears sampled in October 2015 from the Greater Poland Region (maize field location: 50°58′ N, 16°55′ E) in Poland had been previously isolated and identified by Gromadzka et al. [4]. Ten *S. zeae* strains and two *S. strictum* strains were isolated from maize ears collected before harvest in October 2018 (56 samples) from the same maize field location (50°58′ N, 16°55′ E) in the Greater Poland Region (Poland).

### 2.2. Fungal Isolation and Identification

The maize ear samples collected in 2018 were placed in separate paper bags, transported to the laboratory, and dried at room temperature. The maize ears were then hand-shelled, and the separated kernels from each ear were cut with a sterile scalpel and placed in duplicate on agar plates containing a low nutrient SNA medium [11]. A tip of hyphae from each off-white culture was transferred to both potato dextrose agar and synthetic SNA low nutrient agar, and multiple passages were performed to obtain a homogeneous culture. The isolates with cultural and morphological characteristics [8,12] of *Sarocladium* and *Lecanicillium* were molecularly identified as described by Gromadzka et al. [4]. The results of species identification of all *Lecanicillium* strains isolated in 2015 were additionally verified on the basis of sequencing of the large-subunit (LSU, 28S) rDNA region. The results of species identification of all *Sarocladium* strains isolated in both 2015 and 2018 (Table 1) were additionally verified on the basis of sequencing of the large-subunit (LSU, 28S) rDNA region and a fragment of the actin gene (*ACT1*). The 1200-bp target region of rDNA LSU was amplified using the primers LROR [13] and LR6 [14] by PCR annealing at 52 °C and the 370-bp fragment of the actin gene was amplified using primer pair ACT 512-F and ACT 783-R [15] by PCR annealing at 55 °C. Other conditions of PCR and sequencing were the same as those reported by Gromadzka et al. [4]. For identification, the sequences were matched against the nucleotide database using BLASTn (Basic Local Alignment Search Tool) from NCBI [16]. All the LSU and ITS rDNA sequences obtained here as well as reported by Gromadzka et al. [4] were deposited in the NCBI GenBank [16]. The accession numbers are provided in Table 1.

### 2.3. Chemicals and Reagents

Mycotoxin standards (enniatins (Enns) and beauvericin (BEA)) and all chemicals were obtained from Sigma-Aldrich (Steinheim, Germany). HPLC-grade water from our own Millipore water purification system was used for analyses.

### 2.4. Secondary Metabolite Production

*Sarocladium* and *Lecanicillium* isolates studied for the production of secondary metabolites were grown on a rice solid medium. In a 300 mL Erlenmeyer flask, 50 g of commercially available rice kernels and 15 mL of distilled water were added, and the flask was left overnight prior to autoclaving at 121 °C for 30 min. The cooled flasks were inoculated with four disks (4 mm diameter) cut from the advancing edge of a 14-day PDA culture of the fungal isolates. Three replicates were prepared for each fungal isolate. A non-inoculated rice solid medium was used as a negative control. The cultures were incubated at 24 ± 2 °C for 21 days under semi-static conditions (the flasks were manually shaken daily to avoid lumping and anaerobic conditions). After incubation the samples from solid substrate cultures were dried under air and room temperature conditions and ground to fine powder in the grinder.

### 2.5. Sample Extraction and HPLC Analysis of Enns and BEA

Ground material was extracted using 2.5 mL of acetonitrile-methanol-water solution (16:3:1 *v*/*v*/*v*) per gram of sample and then homogenized. The extracts were purified on Florisil columns, and mycotoxins were then estimated using a chromatographic system as described by Jestoi [17].

HPLC analyses of Enns and BEA were performed using a Waters 2695 system equipped with a Waters 2996 Array Detector. The reversed phase column was a C-18 Nova Pak column (3.9 × 150 mm). Samples were eluted with acetonitrile-water (70:30, *v*/*v*) at a constant flow rate of 1 mL min^−1^ for 45 min. Mycotoxins were detected at 205 nm. Enns and BEA were quantified by comparing peak areas of the analyzed samples with the calibration curve of peak areas obtained with authentic mycotoxin standards.

### 2.6. Qualitative Analysis of Selected Metabolites Formed by L. lecanii, S. zeae and S. strictum Isolates Using UPLC/TQD

In order to acquire mass data product-ion spectra of selected metabolites [6,18,19,20], the extracts were analyzed using the Aquity UPLC chromatograph (Waters, Manchester, MA, USA), coupled with an electrospray ionization triple quadrupole mass spectrometer (TQD) (Waters, Manchester, MA, USA). Separation was achieved on a BEH C18 column (100 mm × 2.1 mm i.d., 1.7 µm particle size) (Waters, Manchester, MA, USA) held at 30 °C with the injection volume 3 µL and flow rate 0.3 mL/min. Elution proceeded by means of a linear gradient with solvents A (5 mM ammonium format) and B (acetonitrile) as follows: 0–1 min, 20% B; 1–30 min, 80% B; 30–45 min, 85% B; 45–47 min, 20% B. The mass spectrometer was operated in the full-scan mode in the mass range *m/z* 150–950.

## 3. Results and Discussion

### 3.1. Isolation and Identification or Re-Identification of Sarocladium and Lecanicillium Species Associated with Maize Seeds

Of 26 maize ear samples collected in October 2018 in the Greater Poland Region, six samples (23%) were found to be the source of *Sarocladium* spp. Twelve *Sarocladium* isolates were grown. The morphological and molecular analysis, based on the NCBI GenBank search [16] for sequences homologous over 99–100% similarity to the obtained *ACT1* sequences and LSU and ITS rDNA sequences, resulted in the identification of two species, namely *S. zeae* (10 isolates) and *S. strictum* (two isolates). However, no *Lecanicillium* spp. were isolated from the kernels of these 26 samples. In contrast, in 47% of maize ear samples collected in 2015 from the Greater Poland Region, both *Sarocladium* spp. and *Lecanicillium* spp. were isolated [4]. The 13 previously obtained isolates were molecularly re-identified as *S. zeae* (seven isolates), *S. strictum* (one isolate), and *L. lecanii* (five isolates). Thus, in both 2015 [4] and 2018, *S. zeae* was the most frequently isolated species of the genus *Sarocladium* from maize kernels collected in the Greater Poland Region. The occurrence of *S. zeae* in maize kernels has also been reported in other studies. Initially, Reddy and Holbert [21] described *A. zeae* (*S. zeae*) as the seed-borne causal agent of “black-bundle disease” of maize. Recently, Wicklow et al. [6] and Wicklow and Polling [7] identified *S. zeae* as the beneficial endophyte in maize seeds sampled from USA. Abe et al. [1] evaluated 46 fungal isolates obtained from maize grains with rot symptoms collected from Brazil and found only one *S. zeae* isolate. This isolate was found to produce extracellular hydrolases, namely amylases, cellulases, proteases, and lipases. *Sarocladium zeae* has also been reported as the component of the mycobiota associated with stored maize kernels in China [2,3]. Xing et al. [2] showed that the occurrence of *S. zeae* varies depending on storage time; in samples stored from 6 months to 5 years, *S. zeae* was found only on the seed surface, while in older samples (>9 years), *S. zeae* was isolated from the internal parts of maize seeds. Wang et al. [3] assessed changes occurring in the mycobiome of maize seeds during 12 months of storage and the complex of essential oil treatment using a nonculture-based approach. They demonstrated that *S. zeae* was the predominant species from the genus *Sarocladium*, while its relative abundance varied with storage time or treatment (and was lower in the late stages of storage and after essential oil treatment than during the early stages and in control samples).

*Sarocladium strictum* was the second species of the genus *Sarocladium* found in maize seeds in the present study. This species was represented by only three isolates, namely one (228) from the 2015 season and the remaining two (626 and 605_1) from the 2018 season. In 1995, Tagne examined the occurrence of fungi associated with maize from Cameroon and identified *S. strictum* (as *A. strictum*) as one of the most frequently occurring species in maize seeds. Further research on the interaction between maize and *S. strictum* (as *A. strictum*) isolates from Cameroon led to the detection of the pathogenic nature of this species [22]. *Sarocladium strictum,* as *A. strictum,* was also found to cause disease of strawberry plants [23]. Recent studies on the fungal communities associated with the endosphere of maize plants from India revealed the presence of *S. strictum* in maize nodes [24]. However, the metabarcoding analysis of microbiota in harvested maize samples from Brittany and France showed the occurrence of *S. strictum* in maize stalks [25]. Additionally, Cobo-Díaz et al. [25] showed negative correlations of operational taxonomic units (OTUs) assigned to *S. strictum* to OTUs assigned to *F. oxysporum,* thus suggesting the antagonistic potential of these species and the need for its further validation by using culture-dependent approaches.

Studies complementary to previous research of Gromadzka et al. [4] involving sequence analysis of the LSU rDNA region confirmed five *Lecanicillium* isolates as *L. lecanii* (Zimm.) Zare and Gams (homotypic synonym: *Akanthomyces lecanii* (Zimm.) Spatafora, Kepler and B. Shrestha and *Verticillium lecanii* (Zimm.) Viegas, Zare and Games [26], Kepler et al. [27]; heterotypic synonym *Cordyceps confragosa* (Mains) [28]). *Lecanicillium spp.* are well-known entomopathogenic and nematophagous fungi with antagonistic activity against several plant pathogens [9,10,12,29]. They have also been described as beneficial plant endophytes [30]. *Lecanicillium lecanii* has been reported as a natural endophyte in *Ammophila arenaria* [31], *Dactylis glomerata* [32], *Deschampsia flexuosa* [33], *Elymus farctus* [31], *Laretia acaulis* [34], *Pinus sylvestris* [35], and *Taxus baccata* [36] and the species whose colonization can be induced (triggered) by artificial inoculation. The latter aspect is mainly related to crops such as *Cucurbita maxima* [37], *Gossypium hirsutum* [38], *Solanum lycopersicum* [37], *Phaseolus vulgaris* [37,39], *Pistacia vera* [10], *Triticum aestivum* [37], *Vitis vinifera* [40], and *Zea mays* [37]. To the best of our knowledge, other than the recent study of Gromadzka et al. [4], there are no data on the natural endophytism of *Lecanicillium lecanii* in maize.

### 3.2. Production of Mycotoxins and Other Compounds by the Investigated Sarocladium and Lecanicilium Isolates

**Figure 1:**
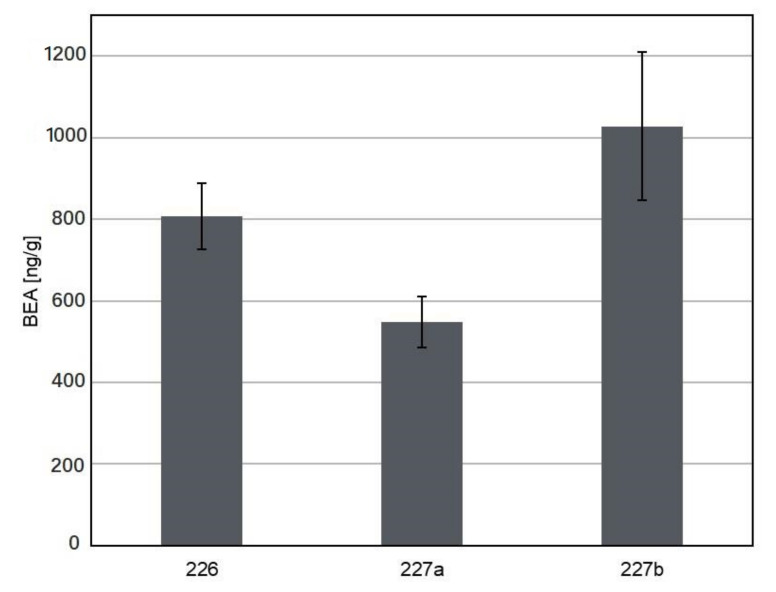
Bauvericin production by *S. zeae* 226, 227a, and 227b isolates.

**Figure 2:**
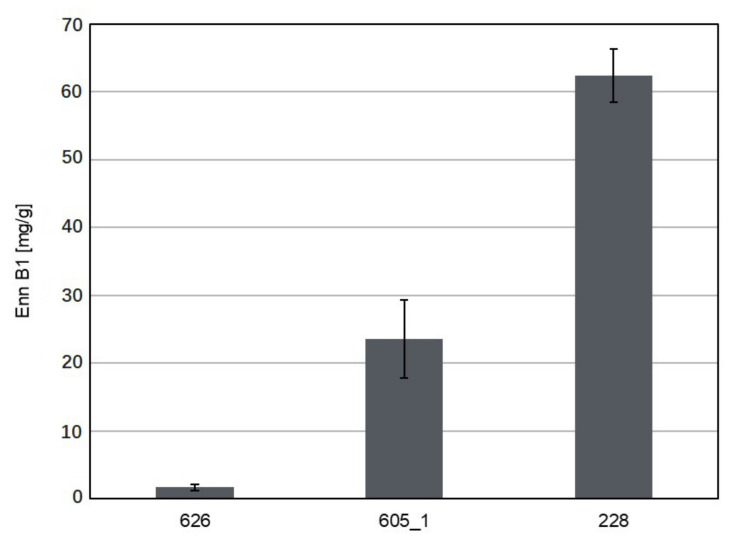
Enniatin B1 production by *S. strictum* 626, 605_1 and 228 isolates.

The isolates of *Sarocladium* and *Lecanicillium* species were assessed for their ability to biosynthesize mycotoxins and other metabolites. Primarily, the most common toxins occurring in Poland were considered: zearalenone; deoxynivalenol; nivalenol; moniliformin; fumonisins; BEA; and Enns A, A1, B, and B1 [41]. The assay on solid substrate (rice) showed that only *Sarocladium* spp. can synthesize two of these mycotoxins, namely BEA and Enn B1. The production of BEA was detected in cultures of all the three tested isolates belonging to *S. zeae*, namely 226, 227a, and 227b (Figure 1). The highest amount of toxin was 1028.39 ng/g for the isolate 227b and the lowest amount was 549.10 ng/g for the isolate 227a. The toxin amount for the third isolate 226 was estimated at 807.68 ng/g. It is interesting to note that all these isolates were collected in 2015, and the isolates 227a and 227b were obtained from seeds of the same maize ears. As observed in the present study, the presence of BEA varied in cultures of *S. zeae* isolates. Thus, this characteristic appeared to be isolate-specific, and not species-specific. The presence of Enn B1 was noted in cultures of all isolates of *S. strictum*. The toxin content of these three isolates ranged from 16.63 to 62.40 mg/g (Figure 2). The present study is the first report on the production of BEA by *S. zeae* and Enn B1 by *S. strictum*. As *S. strictum* is considered to be a plant pathogen [22,23], the ability to produce Enn B1 in isolates from this species is not so controversial as that in the case of *S. zeae*, which is a known plant beneficial endophyte [6,7].

Apart from BEA and Enn B1, a qualitative analysis showed that 20 *Saracladium* and five *Lecanicillium* isolates can synthesize other compounds, including those showing insecticidal and antimicrobial activity [6,18,19,20]. However, the profile of these metabolites was found to be different in the three investigated species (Table 2). Moreover, intraspecies variability was observed in the synthesis of phenopicolinic acid derivatives (vertilecanins), 2-decenedioic acid, 10-hydroxy-8-decenoic acid as well as pyrrocidine A and pyrrocidine B (Table 2). Vertilecanin A was present in all *Sarocladium* isolates, but was not synthesized by *Lecanicillium* spp. Vertilecanin B was not found in any of the tested cultures. Vertilecanin C was produced by one *L. lecanii* isolate 207 and two *S. zeae* isolates: 215 and 217. It is worth noting that although *Lecanicillium* isolates did not synthesize vertilecanin A, they produced its derivative vertilecanin A methyl ester. In addition, vertilecanin A methyl ester was also detected in solid substrate cultures of all *S. strictum* isolates and in 11 *S. zeae* isolates. The studied *L. lecanii* isolates also did not synthesize 10-hydroxy-8-decenoic acid, while this compound was produced by all *S. strictum* and eight *S. zeae* isolates. 2-decenedioic acid was synthesized by two *Lecanicillium* isolates, namely 207 and 213; seven *S. zeae* isolates; and three tested *S. strictum* isolates. Pyrrocidine A and B were identified in cultures of five of the 17 tested *S. zeae* isolates, namely those that were the only ones to produce BEA—226, 227A and 227B as well as in 601 and 614. Moreover, both of these compounds were identified in *S. strictum* 228 isolates, while pyrrocidine A alone was detected as the product of *S. strictum* strain 605-1 and pyrrocidine B as the product of *S. strictum* 626. Pyrrocidine B was also synthesized by *S. zeae* 220, 603, 605, 606, 613 and 652 and *L. lecanii* 207, 213, with no production of pyrrocidine A. However, neither of these two metabolites were detected in the cultures of *S. zeae* 215, 217, 221, 651, 658 and *L. lecanii* 340, 345 isolates.

As shown in the Table 2, the only metabolite formed in the cultures of all the tested *Lecanicillium* and *Sarocladium* isolates was bassianolide. This active insecticidal cyclodepsipepside was previously reported as products of *L. lecanii* [18]. The finding that all *L. lecanii* isolates tested here could produce bassianolide was consistent with the study of Kanaoka et al. [18]. However, for the first time in the present study, these compounds were annotated as metabolites of *S. strictum* and *S. zeae*, which may suggest the insecticidal properties of *S. zeae* and *S. strictum* species. This could also be supported by the currently observed ability of *S. zeae* and *S. strictum* to produce another metabolite with confirmed insecticidal and antibacterial activity, namely vertilecanin A. Verticilian A was also first described as a metabolite of *L. lecanii* ([19], as *Verticillium lecanii*). Interestingly, none of the currently tested *L. lecanii* isolates synthesized vertilecanin A. Soman et al. [19] reported that solid substrate (rice) cultures of *L. lecanii* (as *Verticillium lecanii*) produced four other phenopicolinic acid derivatives as well, namely vertilecanin A methyl ester, vertilecanin B, vertilecanin B methyl ester, vertilecanin C as well as 10-hydroxy-8-decenoic acid and 2-decenedioic acid. Among them, only vertilecanin A methyl ester and vertilecanin C were detected as metabolites of *L. lecanii* in the present study, wherein vertilecanin A methyl ester was detected in the culture of all the tested *L. lecanii* isolates and vertilecanin C was found only in *L. lecanii* isolate 207. Moreover, these (vertilecanin A methyl ester, vertilecanin C) as well as 10-hydroxy-8-decenoic acid were detected in the cultures of the tested here *S. zeae*. Whereas vertilecanin A methyl ester and 10-hydroxy-8-decenoic acid were noted as products of *S. strictum*. This is the first report that vertilecanin A methyl ester, vertilecanin C and 10-hydroxy-8-decenoic acid are described as *S. strictum* and/or *S. zeae* metabolites. However, the insecticidal or antibiotic activity of these compounds has not been demonstrated so far, or even excluded by Soman et al. [19]. Whereas, antimicrobial activity was confirmed for pyrrocidines, mainly pyrrocidin A, which were first isolated from fermentation broth of an unidentified filamentous fungus LL-Cyan426 [20]. He at al. [20] demonstrated the potent antibiotic activity of pyrrocidine A against most Gram-positive bacteria, including the drug-resistant strains, and moderate activity against *Streptococcus pneumonia* as well as yeast *Candida albicans*. Pyrrocidine A and B were then detected in fermentation extracts of *S. zeae* (as *A. zeae*) strains isolated from maize kernels harvested in various locations in the USA and exhibiting antagonistic potential to kernel rotting and mycotoxin producing fungi *Aspergillus flavus* and *Fusarium verticillioides* [6]. As in the present study, also in the work of Wicklow et al. [6], the pyrrocidine profiles showed differences between the *S. zeae* isolates. Wicklow et al. [6] determined the presence of pyrrocidines in ethyl acetate extracts of maize kernel fermentations for 12 out of 13 tested cultures of *S. zeae*. The authors reported that Pyrrocidine A and B were detected in nine of the cultures, while pyrrocidine B alone was detected in three cultures. Subsequent studies by Wicklow et al. [42] confirmed the variation in the distribution of pyrrocidines in the populations of maize endophytes *S. zeae* and supported that the ability of these fungi to form pyrrocidine A and B appeared to be isolate-specific and not species-specific. These findings are consistent with the observations from the present study. Here, apart from *S. zeae*, pyrrocidines were also found in the rice solid cultures of *S. strictum* and *L. lecanii* isolates. Moreover, pyrrocidine A and B were detected in culture of *S. strictum* 226, pyrrocidine A alone was identified in the culture of *S. strictum* 605_1, and pyrrocidine B in the culture of *S. strictum* 626 as well as in the fermentation extracts for 3 (207, 213, 224) cultures of *L. lecanii*.

It is noteworthy that pyrrocidines A and B were discovered to be the metabolites accounting for *S. zeae* antifungal activity against *Aspergillus flavus* and *Fusarium verticillioides* [6,43]. Moreover, Wicklow et al. [42] documented that Pyrrocidine A displayed in vitro activity against major stalk and ear rot pathogens of maize, including *F. graminearum*, *Nigrospora oryzae*, *Stenocarpella* (*Diplodia*) *maydis*, and *Rhizoctonia zeae* as well as seed-infecting colonists of the phylloplane *Alternaria alternata*, *Cladosporium cladosporioides*, and *Curvularia lunata*, which produces a damaging leaf spot disease and seed-rotting saprophyte *Eupenicillium ochrosalmoneum*. Following the suggestion of Wicklow et al. [6,42] that the ability of fungal endophytes to produce pyrrocidine A and B may be important for their interaction with competing microorganisms, it can be assumed that the capacity of maize seed-associated *Sarocladium* and *Lecanicillium* isolates to synthesize these and other antibiotics (bassianolide, vericillian A) observed in present study may also signal their antagonistic potential towards other fungi. It is worth noting that the sources of endophytic isolates tested here were maize ear samples, with (suppression of the *Fusarium* spp. populations) a lower infestation by the populations of *Fusarium* spp., which could be an expression of the antagonistic abilities of these isolates towards *Fusarium* species [4]. It also leads to the presumption that maize ear without *Fusarium* ear rot symptoms can be a source of valuable endophytic fungi with antagonistic potential. Moreover, the analysis of the ability of isolated endophytic fungi to form selected bioactive compounds in solid substrate cultures allowed to identify potential antagonists. This approach may therefore constitute the candidates’ pre-selection strategy for biological control agents screening studies.

## 4. Conclusions

The present study reported the screening of 17 *S. zeae* isolates, three *S. strictum* isolates, and five *L. lecanii* isolates originating from the endosphere of maize seeds for the formation of selected metabolites in solid substrate cultures. BEA, bassianolide, vertilecanin A, vertilecanin A methyl ester, 2-decenedioic acid, 10-hydroxy-8-decenoic acid have not yet been reported to be produced by *S. zeae*. This is also the first study to reveal the ability of *S. strictum* to produce Enn B1, bassianolide, vertilecanin A, vertilecanin A methyl ester, 2-decenedioic acid, 10-hydroxy-8-decenoic acid and pyrrocidine A and B. It should be also noted that pyrrocidine B has never been detected in the rice solid cultures of *L. lecanii* isolates. The production of several bioactive substances, namely toxic and/or insecticidal and antibacterial compounds, in cultures of *S. strictum, S. zeae* and *L. lecanii* suggests that more comprehensive studies and revising the approach to investigate the biological role of fungi inhabiting maize seeds is needed.

## Figures and Tables

**Table 1:** The list of identified or re-identified in present study endophytic fungi isolated from maize ear samples collected in the Greater Poland Region.

Species	Isolate Code	Sampling Year	NCBI GenBank Accession No.	
			ITS	LSU
*Lecanicillium lecanii*	207	2015	MT372981	MT373083
	213		MT372982	MT373084
	224		MT375133	MT375130
	350		MT372983	MT373085
	345		MT372984	MT373086
*Sarocladium strictum*	228	2015	MT375132	MT375131
	626	2018	MT372901	MT374074
	605_1		MT372902	MT374075
*Sarocladium zeae*	215	2015	MT372974	MT373076
	217		MT372976	MT373078
	220		MT372977	MT373079
	221		MT372978	MT373080
	226		MT372979	MT373081
	227A		MT372980	MT373082
	227B		MT372975	MT373077
	601	2018	MT372893	MT374066
	603		MT372894	MT374067
	605		MT372895	MT374068
	606		MT372896	MT374069
	613		MT372893	MT374066
	614		MT372899	MT374072
	636		MT372897	MT374070
	651		MT372898	MT374071
	652		MT372899	MT374072
	658		MT372900	MT374073

**Table 2:** The profiles of selected metabolites formed by *L. lecanii*, *S. zeae* and *S. strictum* isolates grown on a rice solid medium.

Compound		10-hydoksy-8-decenoic acid	2-decenedioic acid	Verttilecanin A	Verttilecanin A methy ester	Verttilecanin C	Pyrrocidine A	Pyrrocidine B	Enns B1	BEA	Bassianolide
Species	Isolate Code										
*L. lecanii*	207	-^1^	+^2^	-	+	+	-	+	-	-	+
	213	-	+	-	+	-	-	+	-	-	+
	224	-	-	-	+	-	-	+	-	-	+
	350	-	-	-	+	-	-	-	-	-	+
	345	-	-	-	+	-	-	-	-	-	+
*S. strictum*	228	+	+	+	+	-	+	+	+	-	+
	626	+	+	+	+	-	-	+	+	-	+
	605_1	+	+	+	+	-	+	-	+	-	+
*S. zeae*	215	+	-	+	+	+	-	-	-	-	+
	217	+	+	+	+	+	-	-	-	-	+
	220	-	-	+	+	-	-	+	-	-	+
	221	+	+	+	+	-	-	-	-	-	+
	226	+	+	+	+	-	+	+	-	+	+
	227A	+	+	+	+	-	+	+	-	+	+
	227B	+	-	+	+	-	+	+	-	+	+
	601	-	-	+	+	-	+	+	-	-	+
	603	+	-	+	-	-	-	+	-	-	+
	605	-	+	+	+	-	-	+	-	-	+
	606	+	-	+	-	-	-	+	-	-	+
	613	-	-	+	+	-	-	+	-	-	+
	614	-	-	+	-	-	+	+	-	-	+
	636	-	+	+	+	-	-	+	-	-	+
	651	-	-	+	-	-	-	-	-	-	+
	652	-	+	+	-	-	-	+	-	-	+
	658	-	-	+	-	-	-	-	-	-	+

’-’^1^ Compound not detected in culture samples; ‘+’^2^ compound detected in culture samples.

## Data Availability

National Centre for Biotechnology Information, https://www.ncbi.nlm.nih.gov/genbank/.
